# Defining the genetic components of callus formation: A GWAS approach

**DOI:** 10.1371/journal.pone.0202519

**Published:** 2018-08-17

**Authors:** Gerald A. Tuskan, Ritesh Mewalal, Lee E. Gunter, Kaitlin J. Palla, Kelsey Carter, Daniel A. Jacobson, Piet C. Jones, Benjamin J. Garcia, Deborah A. Weighill, Philip D. Hyatt, Yongil Yang, Jin Zhang, Nicholas Reis, Jin-Gui Chen, Wellington Muchero

**Affiliations:** 1 Plant Systems Biology Group, Biosciences Division, Oak Ridge National Laboratory, Oak Ridge, Tennessee, United States of America; 2 Department of Forest Ecosystems and Society, Oregon State University, Corvallis, Oregon, United States of America; 3 The Bredesen Center for Interdisciplinary Research and Graduate Education, University of Tennessee, Knoxville, Tennessee, United States of America; 4 School of Forest Resources and Environmental Science, Michigan Technological University, Houghton, Michigan, United States of America; 5 Computational Biology Group, Biosciences Division, Oak Ridge National Laboratory, Oak Ridge, Tennessee, United States of America; 6 Oak Ridge Associated Universities, Oak Ridge, Tennessee, United States of America; Clemson University, UNITED STATES

## Abstract

A characteristic feature of plant cells is the ability to form callus from parenchyma cells in response to biotic and abiotic stimuli. Tissue culture propagation of recalcitrant plant species and genetic engineering for desired phenotypes typically depends on efficient *in vitro* callus generation. Callus formation is under genetic regulation, and consequently, a molecular understanding of this process underlies successful generation for propagation materials and/or introduction of genetic elements in experimental or industrial applications. Herein, we identified 11 genetic loci significantly associated with callus formation in *Populus* trichocarpa using a genome-wide association study (GWAS) approach. Eight of the 11 significant gene associations were consistent across biological replications, exceeding a chromosome-wide–log10 (p) = 4.46 [p = 3.47E−05] Bonferroni-adjusted significance threshold. These eight genes were used as hub genes in a high-resolution co-expression network analysis to gain insight into the genome-wide basis of callus formation. A network of positively and negatively co-expressed genes, including several transcription factors, was identified. As proof-of-principle, a transient protoplast assay confirmed the negative regulation of a Chloroplast Nucleoid DNA-binding-related gene (Potri.018G014800) by the LEC2 transcription factor. Many of the candidate genes and co-expressed genes were 1) linked to cell division and cell cycling in plants and 2) showed homology to tumor and cancer-related genes in humans. The GWAS approach based on a high-resolution marker set, and the ability to manipulate targets genes *in vitro*, provided a catalog of high-confidence genes linked to callus formation that can serve as an important resource for successful manipulation of model and non-model plant species, and likewise, suggests a robust method of discovering common homologous functions across organisms.

## Introduction

Callus arises in plants through the cellular reprogramming of parenchyma cells [1], leading to a disorganized amorphic mass of rapidly dividing cells. Callus induction is triggered by variations in endogenous plant hormone levels that occur in response to physical or chemical stimuli [2, 3]. There are several regulatory cascades and pathways that lead to cellular reprogramming, including a cytokinin-based route, an auxin-based route and a wound-induced route [3]. Wound-induced cellular reprogramming can occur due to bacterial, viral, and/or insect attack, as well as physical abrasion. *In vivo* callus formation has been generally observed across all higher plant genera. It was first reported in excised stem tissue of poplar, which was subsequently induced to form roots and shoots [4]. Callus induction is the basis of many *in vitro* plant regeneration protocols [5] that are prerequisites for genetic engineering and genome editing [6]. Moreover, plant callus formation shares similar anatomical and physiologic features with human tumor formation [7], highlighting the value of understanding the underlying mechanisms callus formation across the tree of life. Fully defining the genetic components of callus induction and formation is therefore of broad general interest to plant and animal biologists.

Individual species, as well as genotypes within a species, vary in their ability to form callus. Despite significant progress in the field [8, 9], some commercially important plant species or genotypes within species often lack effective *in vitro* culture and callus induction protocols. This is particularly true for non-domesticated *Populus*, and without this capacity, creation of transgenic plants is difficult. Since callus induction and proliferation is under genetic control and regulation, identifying the genes and regulatory elements that control callus formation has the potential to facilitate the development of *in vitro* systems in recalcitrant plant species. In addition, identification of these genes or regulatory elements could also provide insights into uncontrolled proliferation of cell division in many organisms, including tumor formation in animal systems. We hypothesize that the developmental regulatory pathways governing the maintenance of cell differentiation and growth are strongly genetically controlled and that high-resolution genome-wide association and co-expression network analyses can define the genetic components of callus formation.

Genome-wide association studies (GWAS) have been used successfully in humans, as well as domesticated plants and animals, to identify genomic regions linked to various phenotypic traits. Relying on high-density single nucleotide polymorphism (SNP) and insertion/deletion (INDEL) markers, causal alleles and single amino acid substitutions have been identified using GWAS approaches in *Populus*, leading to 1) validation of gene-to-phenotype associations [10, 11], 2) cloning causal alleles [12], and 3) stable and transient transformation [13]. In this study, we leveraged an 8.2 million SNP library to interrogate a 280-genotype population of *Populus trichocarpa* in order to identify genes associated with callus formation. We show that there are eight genes highly associated with this process and that these genes are co-expressed within a network of other genes related to cell division and cell cycling. We also provide evidence that these genes are unique relative to those genes reported in ectopic *Arabidopsis* callus formation. Our findings also show homology-based evidence for similar genetic mechanisms in human tumor and cancer associated genes.

## Materials and methods

### Plant materials

From within 1084 genotypes of *Populus trichocarpa* contained in the GWAS population [14, 15] we tested callus induction in 280 genotypes. To avoid potential bias in allele frequencies, these genotypes were selected to represent the latitudinal gradient across the natural range of this species in the Pacific Northwest of North America. Global Position Systems (GPS) co-ordinates recorded when each genotype was originally collected were used to uniformly sample across river systems in this range [16]. Clonal replicates of each genotype were grown in the greenhouse for three months prior to sampling leaf tissue for explant establishment. Each genotype had been re-sequenced to a minimum of an 18x depth and a SNP library with 8.2 million SNPs was available for the GWAS analyses [https://cbi.ornl.gov/data]. Whole-genome resequencing, alignment of Illumina short reads to the reference *P*. *trichocarpa* genome, SNP calling and data curation parameters are fully described by Evans et al. [14].

### Callus induction

Multiple fully expanded leaves were collected from each genotype and surface disinfested using 1% (v/v) Tween-20 solution for 3 to 5 min, 70% (v/v) ethanol for 1 min, and 10% bleach solution (5.25% sodium hypochlorite) for 10 min, followed by 3 rinses with sterile, distilled water [17]. Explants were aseptically cut from leaves using a 1 cm^2^ diameter cork borer and placed adaxial-side up on medium previously proven successful for callus induction of *Populus trichocarpa* [18]. Specifically, a Murashige and Skoog (MS) medium [19] was supplemented with 0.5 μM 6-benzylaminopurine (BA), 0.5 μM zeatin, 5 μM naphthaleneacetic acid (NAA), 5 μM 2,4-D, and 1.28 mM 1-morpholinoethanesulfonic acid (MES), adjusted to a pH of 5.8, and solidified using 0.3% Phytoagar and 0.1% Gelrite. Midveins within the leaf explants were targeted as explants due to their organogenic potential. Three replications with 12 leaf disks per plate per replication were initiated. Cultures were then incubated for 4 weeks under constant dark at 25˚C.

A second callus induction experiment was conducted using the seven genotypes each with the most and least prolific callus formation. Following the same protocol described above, leaf explants were cultured on media with varying phytohormone levels. Combinations of high and low cytokinin to auxin were tested: high cytokinin/low auxin with 5 μM BA + 0.5 μM TDZ + 0.5 μM NAA; high cytokinin/medium auxin with 5 μM BA + 0.5 μM TDZ + 1 μM NAA; high cytokinin/high auxin with 5 μM BA + 0.5 μM TDZ + 5 μM NAA; low cytokinin/low auxin with 1 μM BA + 0.5 μM TDZ + 0.5 μM NAA; low cytokinin/medium auxin with 1 μM BA + 0.5 μM TDZ + 1 μM NAA; low cytokinin/high auxin with 1 μM BA + 0.5 μM TDZ + 5 μM NAA. Three replications with 12 leaf disks per plate per replication were examined. Cultures were incubated for 4 weeks under constant dark at 25˚C.

### Callus rating

The number of explants forming callus was counted and scored based on the amount of callus formed. Callus formation was recorded as a percent of the 12 explants per replicate forming callus. Callus ratings score were assigned as follows: 0 for no callus formation, 1 for compact callus, 2 for green friable callus, and 3 for white friable callus. Callus organogenic potential is known to vary by callus appearance [18], with white friable callus leading to greater shoot induction potential. Location of callus formation on the explant was also noted as initiating from the midvein and/or from the cut edge.

### Analysis of variance

A two-way ANOVA, with genotype (G), replication (R) and GxR interaction as random effect sources of variation, was used to test for significant differences among genotypes in callus formation and callus rating (p≤0.05). Broad-sense heritability was calculated as the variance due to genotype divided by the summation of the error variance plus the genotype variance. Heritability was only calculated when there were significant genotype effects. A one-tailed t-test (p≤0.05) was used to test difference among hormone treatments in the second callus induction experiment.

### Genome-wide association test

To determine genetic loci associated with callus formation or callus rating, we used the EMMAX algorithm, with kinship as the correction factor for genetic background effects [20], to compute genotype-to-phenotype associations using 8.2 million SNPs with minor allele frequencies ≥0.05 as described by Zhang et al. (2018) [21]. Callus formation and callus rating candidate genes were identified based SNP association which exceeded the chromosome-wide–log10 (*p*) = 4.46 [*p* = 3.47E−05] Bonferroni-adjusted significance threshold. GWAS tests were run independently by replicates and only those associations that were significant across all three replicates are reported here.

### Gene Atlas analysis

Gene Atlas data [22] for four callus formation and four callus rating genes were collected from Phytomine database integrated in Phytozome (v.11.0) with FPKM value [23, 24]. The log2 scaled FPKM from a total of 24 different tissue types or conditions were subjected to ‘gplots’ of R package and summarized in heat maps [25].

### Gene Co-expression network construction and gene ontology enrichment

Gene Atlas data across seven tissues was also used to calculate Pearson correlation coefficients between the expression profiles of all pairs of genes using the mcxarray and mcxdump programs from the MCL-Edge software package [26] available from: http://micans.org/mcl/. Correlation were calculated in a parallel fashion making use of the Parallel::MPI::Simple Perl module available on the Comprehensive Perl Archive Network (CPAN) at [www.cpan.org]. A respective 0.8, and -0.8 Pearson threshold was applied and subnetworks of genes that co-express (positive or negative) with the eight candidate genes identified by GWAS were created and visualized in Cytoscape [27].

### *Arabidopsis* callus orthologs

*Arabidopsis*-based microarray expression data was obtained from GSE29543 [www.ncbi.nlm.nig.gov/geo/], probes were mapped to the Affymetrix ATH1-121501 *Arabidopsis* annotation V35, expression was normalized using robust multi-array averaging (RMA) [https://www.ncbi.nlm.nih.gov/pubmed/12582260] and then Linear Models for Microarray and RNA-Seq Data (Limma) [https://link.springer.com/chapter/10.1007/0-387-29362-0_23] was used to calculate differential expression. Time points of 12 h, 24 h, 48 h and 96 h of callus induction were compared to 0 h, representing establishment of shoot explants introduced to callus inducing media. A heat map was then constructed based on fold-change values from *Arabidopsis* genes that were significantly differentially expressed in shoot (adjusted p-value≤0.05) in at least one comparison and that were orthologs to *Populus* genes associated with callus formation.

### Transient overexpression in *Populus* protoplast and quantitative RT-PCR (qRT-PCR)

Protoplasts from hybrid poplar 717 (*Populus tremula* X *alba*) leaves were isolated and subsequently transfected as previously described [13]. The full-length CDS of LEC2 (Potri.004G045800) was determined according to the sequence information available at Phytozome [https://phytozome.jgi.doe.gov/]. The CDS of LEC2 was introduced into the pENTR/D-TOPO vector (Life Technologies), and subsequently transferred into a Gateway destination vector via LR reaction. The Gateway destination vector was constructed by amplifying the 35S promoter, the Gateway cassette and the Tnos terminator from pGWB502 [28], using primers 5’-ATGGTACCTGAGACTTTTCAACAAAGGGTA-3’ and 5’-ATAAGCTTGATCTAGTAACATAGATGACAC-3’, was subcloned into the pUC19 vector using restriction enzymes KpnI and HindIII.

Total RNA from transfected and control *Populus* protoplasts was extracted using the Spectrum Plant Total RNA isolation kit (Sigma). One μg of total RNA were reversely transcribed to cDNA using RevertAid Reverse Transcriptase (Thermo Fisher Scientific). qRT-PCR was performed using Maxima SYBR Green/ROX qPCR Master Mix (Thermo Fisher Scientific). *Populus* Ubiquitin (UBQ10b) was used as an internal control for normalizing the relative transcript level. All PCR reactions were completed with at least three replicates. The primers used for qRT-PCR are listed in S1 Table.

## Results

### Callus formation and rating is genotype dependent

**Fig 1:**
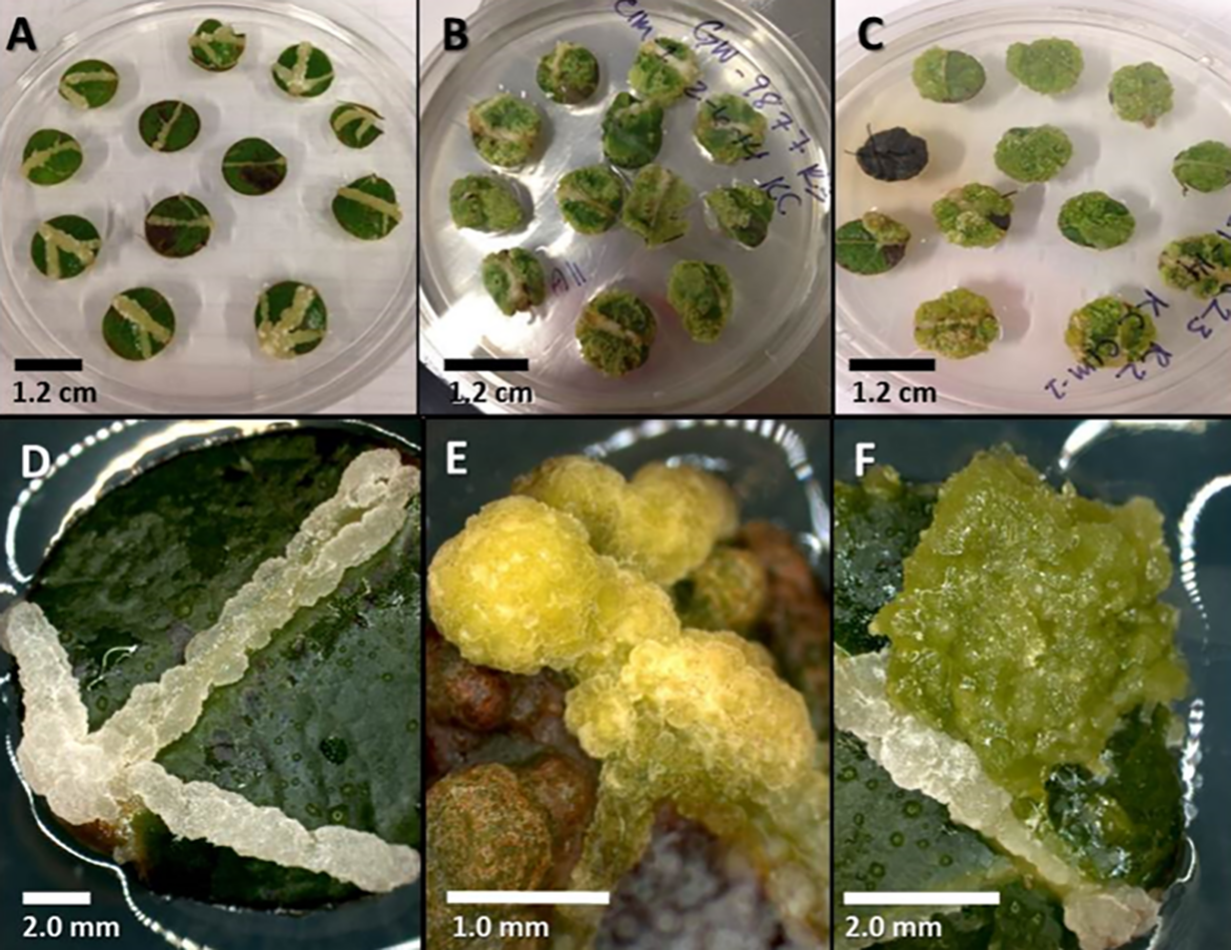
Callus formation on *Populus* leaf disc explants after 30 days on a callus induction medium. (A) 12 replicate leaf disk explants with callus along the midrib, (B) 12 replicate leaf disk explants with callus across the explant, (C) 12 replicate leaf disk explants with callus along the cut margin, (D) white friable callus along the midrib, (E) light green compact callus, and (F) green friable callus.

Among the 280 *P*. *trichocarpa* genotypes tested for callus induction, 21 genotypes produced no callus and 30 genotypes formed callus from 100% of their explants (Fig 1). The mean callus formation frequency across all genotypes was 53%+1.9% (mean+s.e.). Among those genotypes that did form callus; the mean callus rating was 1.3+0.05, with only 49 genotypes averaging a rating of 2.5 or higher. In total, 101 genotypes had a mean callus rating score of 1.0 or greater. Callus formation and callus rating were positively correlated with r^2^ = 0.77. Of the explants that formed callus, 73% initiated from the midrib and 25% formed callus along the cut edge of the leaf explant. Genotype had a significant effect on callus formation (F_279, 558_ = 7.16, p-value = 4.28E-86) and callus rating (F_279, 558_ = 6.56, p-value = 5.42E-79). Broad-sense heritability for callus formation was h^2^ = 0.67 and heritability for callus rating h^2^ = 0.65.

### Candidate genes associated with callus formation and rating

**Fig 2:**
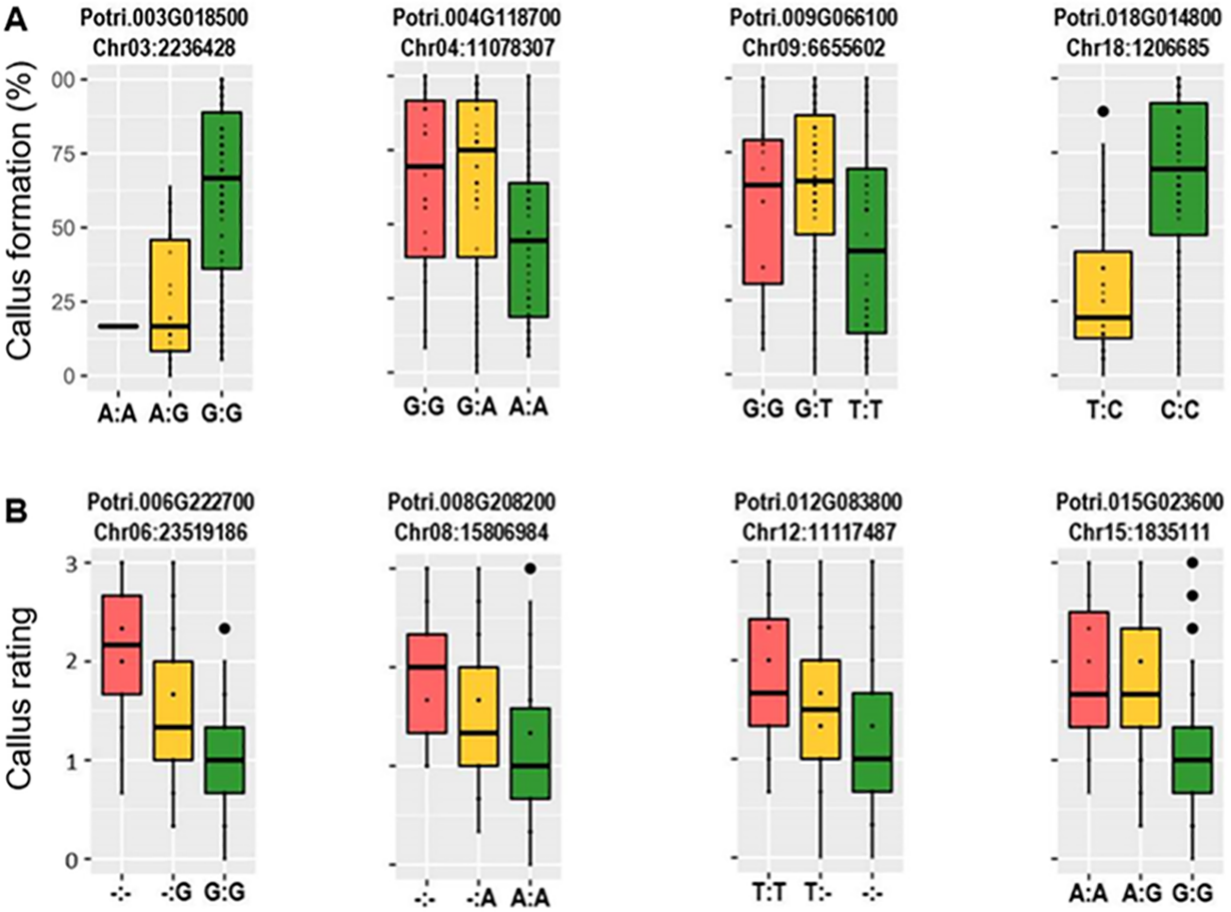
Box plots for (A) callus formation and (B) callus rating score from a genome-wide association test. In each panel, the homozygous rare (*i*.*e*., less common) alleles are displayed to the left in the red box, the heterozygous genotypes in the yellow box and the homozygous common alleles in the green box.

Among the 11 significant GWAS associations (S2 Table), seven were significant for callus formation and four were selected for further study based on their repeated occurrence across biological replicates (Fig 2A)—Potri.003G018500, Potri.004G118700, Potri.009G066100, and Potri.018G014800 (p-value = 9.90E-08, 4.27E-07, 9.72E-08 and 3.83E-07, respectively). Potri.003G018500 encodes a SOK1 kinase containing a Testis-complex protein 11 motif that is highly expressed in early male flower development (S1 Fig) and co-expressed with Potri.015G078200—a gene of unknown function and Potri.016G082400—a kinesin motor protein-related protein (r^2^ = 0.89 and r^2^ = 0.86, respectively). Potri.004G118700, is a targeting protein for XKLP2 and is highly expressed in fully opened buds, immature leaves and root tips (S1 Fig) and is co-expressed with numerous genes including: Potri.002G080000—a mitotic-specific cyclin-B protein, Potri.016G033000—a cyclin G protein, Potri.017G081000—a tubulin, Potri.005G257500—a cyclin-dependent kinase and Potri.005G258300—a spindle checkpoint protein (r^2^ = 0.99, r^2^ = 0.98, r^2^ = 0.98 and r^2^ = 0.96, respectively). Potri.009G066100 encodes a mitogen-activated protein kinase (MAPK3) which is highly expressed in roots under high nitrogen and urea (S1 Fig) and is co-expressed with many genes including Potri.008G082100—a cell cycle control protein and Potri.016G009700—a scarecrow-like protein (r^2^ = 0.92 and r^2^ = 0.92, respectively). Finally, Potri.018G014800 is Chloroplast Nucleoid DNA-binding-related gene (CNDbr) which includes an aspartyl protease family protein domain, and is highly expressed in young leaves, stem nodes and internodes, root tips and in roots under high ammonia and nitrogen (S1 Fig).

Among the 11 significant GWAS associations for callus rating, four were significant across all biological replicates (S2 Table)—Potri.006G222700, Potri.008G208200, Potri.012G083800 and Potri.015G02360 (p-value = 1.61E-07, 8.15E-07, 5.44E-07 and 6.53E-07, respectively) (Fig 2B). Potri.006G222700 is a gene of unknown function and is expressed in late development female flowers and dormant buds and is found in *Salix purpurea*, *Theobroma cacao*, and *Manihot exculenta* with >80% amino acid similarity (S1 Fig). Potri.008G208200, a RALF-LIKE protein 22, is highly expressed in early developing male flowers (S1 Fig). Potri.012G083800, a RPD3 histone deacetylase protein, is moderately expressed in dormant buds (S1 Fig) and is co-expressed with multiple genes including Potri.010G213700—a LEUKOCYTE RECEPTOR CLUSTER MEMBER 8 protein and Potri.009G137200 –a transcriptional coactivator CAPER RRM superfamily protein (r^2^ = 0.93 and r^2^ = 0.92, respectively). Potri.015G023600, a second gene of unknown function, is moderately expressed in multiple tissues and is found in *S*. *purpurea* with >95% amino acid similarity (S1 Fig). Interestingly, Potri.015G023600 contains a non-annotated RNA transcribed from the sequence between the 4th and 5th exons. This RNA is found in various tissues and contains no known domains or motifs (data not shown). Potri.015G023600 is co-expressed with several zinc-finger proteins (r^2^ = 0.85–0.91) and Potri.003G195400 encodes an ARMADILLO repeat-containing protein (r^2^ = 0.92).

By examining the boxplots for each of the eight candidate genes, we found that the rare allele (defined as the less frequent allele in the test population and depicted in the left column of each boxplot) for Potri.003G018500 and Potri.018G014800 lead to reduced callus formation, whereas the rare allele for Potri.004G118700 and Potri.009G066100 lead to increased callus formation (Fig 2A). Interestingly, genotypes with homozygous rare alleles for Potri.003G018500 and Potri.018G014800 were not found in the tested population, suggesting that this condition may be lethal. Callus rating scores were all higher for the rare alleles for Potri.006G222700, Potri.008G208200, Potri.012G083800, and Potri.015G023600 (Fig 2B). Three of the candidate genes identified via the GWAS analysis for callus rating were associated with small frameshift INDELs.

### Callus formation validation

Callus formation *in vitro*, which is dependent on the plant source tissue and genetic background, varies with the concentration and ratios of added exogenous phytohormones to the plant media [29]. We therefore hypothesize that the *Populus* genotypes with the alleles associated with increased callus formation will consistently perform better in the different phytohormone treatments while those genotypes with the alleles associated with reduced callus formation will maintain reduced callus formation capacity due to their genetic background. To validate the initial callus formation experiment, and to leverage the information contained in the GWAS analyses, we initiated an independent phytohormone treatment experiment based on six phytohormone combinations and seven genotypes that initially produced abundant callus with higher rating scores and contained the alleles associated with increased callus formation (*i*.*e*., BESC-18, BESC-233, BESC-823, GW-9795, GW-9877, GW-9920, and HOMB-21-2) and seven genotypes that had low occurrence of callus formation (*i*.*e*., BESC-100, BESC-106, BESC-352, BESC-856, BESC-89, GW-9904, and YALD-27-2). These genotypes were selected specifically because they contained high impact mutations (*i*.*e*., frameshifts or premature stop codons) predicted by genotype resequencing data using SnpEff [https://phytozome.jgi.doe.gov/] in one or more of the significant loci identified in the GWAS results. Based on a one-tailed t-test, there were significant differences between the high callus producing genotypes and the low callus producing genotypes across all phytohormone combinations tested (t = 3.70, p = 2.03E-3). The abundant callus forming genotypes also had consistently higher callus rating scores across all phytohormone combinations, with the exception of genotypes BESC-18 and GW-9877 (Table 1).

**Table 1:** Callus rating scores (0–3) for seven *Populus* genotypes predicted to have high callus formation potential and seven *Populus* genotypes with low callus formation potential. Dash (-) indicates callus cultures that were lost due to contamination.

Genotype	Predicted Status	High cytokinin, Low auxin	High cytokinin, Med. auxin	High cytokinin, High auxin	Low cytokinin, Low auxin	Low cytokinin, Med. auxin	Low cytokinin, High auxin	Mean^1^
BESC-18	High	1.5	2.0	-	1.5	1.0	2.5	1.90
BESC-233	High	3.0	3.0	-	-	1.0	2.0	2.75
BESC-823	High	2.5	2.5	-	2.0	-	2.0	2.25
GW-9795	High	3.0	2.0	1.5	2.0	2.0	2.5	2.00
GW-9877	High		1.0	2.0	1.0	2.0	1.5	1.50
GW-9920	High	2.5	2.5	2.5	-	3.0	2.5	2.60
HOMB-21-2	High		2.0	2.0	1.5	2.0	-	1.88
**Mean**		2.3	2.1	2.0	1.6	2.2	3.3	2.09
BESC-100	Low	2.0	2.5	1.5	2.0	2.0	2.0	2.00
BESC-106	Low	1.5	0.5	1.0	1.0	1.5	0.5	1.00
BESC-352	Low	-	1.5	0.5	2.0	1.5	2.0	1.50
BESC-856	Low	0.5	0.0	0.0	1.0	-	1.0	0.50
BESC-89	Low	0.0	2.0	-	1.5	-	2.0	1.38
GW-9904	Low	0.0	0.0	0.5	0.5	0.5	-	0.30
YALD-27-2	Low	0.0	0.0	0.0	0.5	0.0	0.0	0.08
**Mean**		0.7	0.9	0.6	1.2	1.1	1.3	0.96
**Overall Mean**		1.5	1.6	1.2	1.4	1.7	1.8	

^1^Overall callus rating means between genotypes with high versus low callus formation potential was significant based on a t-test for two samples assuming unequal variances at p≤0.05.

### Callus formation genes co-expressed with genes related to cell differentiation and growth

**Fig 3:**
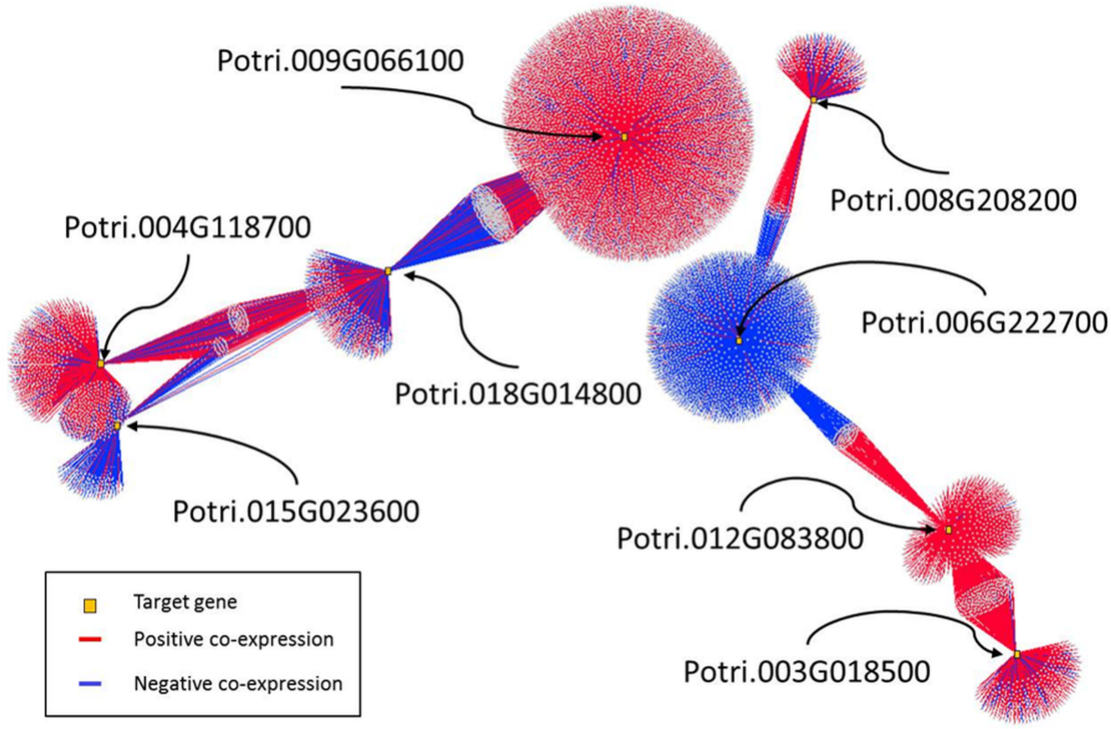
Co-expression networks for the eight-significant genome-wide association loci related to callus formation and callus rating in *Populus*. Red edges indicate a positive co-expression at r≥0.9 and blue edges indicate negative co-expression at r≤-0.9.

Candidate genes from the GWAS were used as query in a co-expression of expressed genes in the Gene Atlas dataset (Fig 3). The genome-wide co-expression network revealed that among the eight candidate genes, Potri.006G222700 and Potri.015G023600, were generally negatively co-expressed with their respective neighboring gene nodes in the co-expression network (S3–S5 Tables); while Potri.003G018500, Potri.012G083800, Potri.008G208200, Potri.009G066100, and Potri.004G118700, were overwhelmingly positively co-expressed with their respective neighboring gene nodes in the co-expression network (S6–S8 Tables). Potri.015G023600 and Potri.004G118700 were the only two candidate genes that were co-expressed with each other (S6 Table). These two genes were also consistently and commonly negatively or positively co-expressed with 332 other genes, respectively, including 35 putative transcriptional regulators, 44 protein kinases, and 10 cell-cycle-related genes (S4 Table). Potri.006G222700 and Potri.012G083800 were also in a reciprocal co-expression network involving 77 genes including KNUCKLES (KNU) that mediates the repression of WUSCHEL (WUS), a floral meristem determinacy gene (homologous to AT5g14010), a phosphoribosyl transferase family protein involved in cellular biosynthesis (homologous to AT2g35390) and two genes related to microtubule organization (S4 Table). A group of genes which co-expressed simultaneously with three candidate genes (Potri.004G118700, Potri.015G023600 and Potri.018G014800) were identified (S6 Table). Generally, Potri.015G023600 was negatively co-expressed with this set of genes, while Potri.004G118700 and Potri.018G014800 were positively co-expressed with this set of genes. This subnetwork involving co-expression with Potri.004G118700, Potri.015G023600 and Potri.018G014800 includes genes related to arrested embryo development (Potri.010G020600, homologous to AT3g06350 (*MEE32*)) and a microtubule-binding protein (Potri.005G033200, homologous to AT3g05330 (*TANGLED1*)). In addition, the co-expressed gene neighborhoods for Potri.004G118700 and Potri.015G023600 were enriched for cell cycle and microtubule formation genes, whereas the neighborhood between Potri.006G222700 and Potri.008G208200 contained quite a few transcription factors and genes of unknown function. The distinctive positive and negative co-expression subnetworks (Fig 3) strongly indicate tight orchestration of gene expression related to callus induction and repression.

Analysis of differential expression in *Arabidopsis thaliana* callus formation data from GEO (GSE29543) revealed that five orthologs to our candidate *Populus* genes were significantly differentially expressed in shoot callus formation in *Arabidopsis* (S2 Fig). Two of these orthologs (orthologous to Potri.004G118700 and Potri.012G083800) were upregulated during callus formation, while two alternate orthologs (Potri.009G066100 and Potri.003G018500) were downregulated during callus formation, again suggesting a network of genes that induce or repress callus formation.

Interestingly, orthologs of genes reported in *Arabidopsis* transgenesis experiments do occur in our co-expression network. Two *LBD16* genes, Potri.005G221900 (orthologous to AT2g42430) and Potri.002G041200 (orthologous to AT2g23380), are negatively co-expressed with Potri.009G066100, along with Potri.002G044100 (orthologous to AT1g231970, *LEC1*, [30]), Potri.002G071200 (orthologous to AT5g49720, TSD1, [31]), Potri.005G188500 (orthologous to AT2g30580, *BM1A*, [32]), and Potri.011G054000 (orthologous to AT1g28300, *LEC2*, [33]) (S3 Fig). Potri.011G054000 is also negatively co-expressed with our candidate gene Potri.018G014800. A paralog of Potri.011G054000, Potri.004G045800 is positively co-expressed with both Potri.003G018500 and Potri.012G083800. Potri.007G012100 (orthologous to AT2g17950, *WUS*, [34]) is positively co-expressed with Potri.012G083800 and negatively co-expressed with Potri.012G083800. Potri.005G140200 (orthologous to AT2g23380, *CLF*, [35]) was negatively co-expressed with Potri.015G023600.

**Fig 4:**
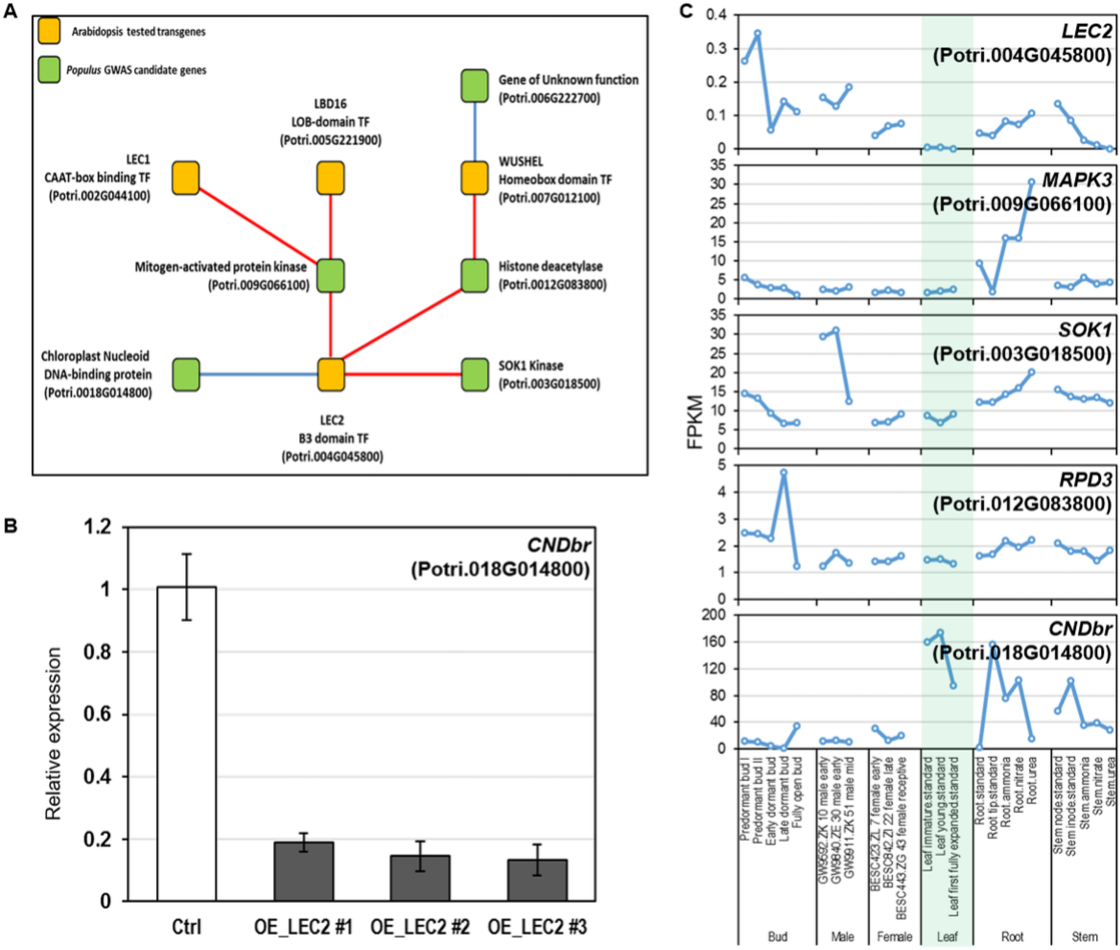
A) Combined genome-wide association results and *Populus* co-expression analyses, with Populus homologs of *Arabidopsis*-tested transcription factors, in a proposed regulatory network. Gold boxes are *Populus* homologs of *Arabidopsis*-tested transcription factors; green boxes are GWAS identified *Populus* genes associated with callus formation. Red edges indicate positive co-expression, blue edges indicate negative co-expression. B) The CNDbr, which negatively co-expressed with LEC2, was down-regulated in *Populus* leaf protoplasts when overexpressing LEC2. The other three genes (SOK1, MAPK3, and RPD3), positively co-expressed with LEC2, were not detected by qRT-PCR. Ctrl refers to the endogenous expression level of CNDbr in protoplasts while OE_LEC2 refers to the expression level of CNDbr when LEC2 was overexpressed in 3 independent replicates. The expression level of CNDbr was normalized to the ubiquitin internal control. C) Expression patterns of five selected genes in co-expression network. LEC2 has extremely low abundance in leaves while CNDbr was highly expressed in leaves. SOK1, MAPK3, and RPD3 showed low abundances in leaf tissues.

Co-expression of the candidate genes from our study, with orthologs of genes functionally validated in callus formation in the model plant *Arabidopsis*, provides support for our GWAS approach used to identify genes targets involved in this process in *Populus*. Based on both GWAS results and the co-expression analyses of the *Populus* candidate genes with the tested and published *Arabidopsis* transgene results, we propose a regulatory gene network for callus formation (Fig 4A). Within this regulatory network, the gene encoding the transcription factor LEC2 containing the B3 domain showed either a positive or negative correlation to 4 of the 8 candidate GWAS genes identified in this study and may function as a hub gene control downstream expression of other transcription factors and kinases. Using a transient expression system in protoplast and quantitative RT-PCR (qRT-PCR), we examined the ability of LEC2 to negatively regulate the expression of the Chloroplast Nucleoid DNA-binding-related gene (Potri.018G014800, CNDbr) and positively regulate the expression of SOK1, MAPK3 and RPD3 (Potri.003G018500, Potri.009G066100 and Potri.012G083800, respectively). We found that when LEC2 was constitutively overexpressed, CNDbr was significantly repressed (Fig 4B); however, the three positively regulated candidate GWAS genes which also showed low abundance in leaf tissue, were not detected in the transient expression assay (Fig 4C).

## Discussion

Completely defining the genetic components of cell de-differentiation and callus formation is of broad interest and application. Induction of pluripotency has implications in understanding orchestrated cell proliferation as well as normal tissue and organ development. Here we identified eight genes associated with callus formation or callus rating in *Populus*. These eight loci were distributed across the *Populus* genome on chromosomes III, IV, XI, VIII, IX, XII, XV and XVII. All eight loci have paralogs within the *Populus* genome that were the result of the Salicoid duplication event that occurred approximately 64 mya [36]. None of the paralogs showed significant association with callus formation or callus rating, suggesting that subfunctionalization may have occurred in these gene lineages. Among the eight significant associations, Potri.004G118700, Potri.008G082100 and Potri.009G066100 are co-expressed with genes annotated with functions related to cell division and cell differentiation; Potri.012G083800 is known to affect chromatin remodeling and an ortholog of Potri.008G208200 has been reported to be potentially involved in callus formation in sugarcane [37]. In total, the evidence suggests that there are networks of genes that tightly regulate the cell division and cell differentiation cascade controlling callus formation.

Potri.004G118700, LEC2, may function as upstream regulator of several genes related to callus formation, including Potri.003G018500, Potri.009G066100, Potri.012G083800 and Potri.018G014800 (Fig 4A). Specifically, Potri.003G018500, a SUPPRESSOR OF KINASE (SOK1) kinase, belongs to the STE20/SPS1/GC kinase family (Pfam PF05794), and there are multiple frameshift mutants at this locus within the GWAS population that cause a gained stop codon at position Chr03:2242626 bp. STE20 kinases in general are thought to regulate MAPK cascades, including several eukaryotic T-complex protein 11 (Tcp11)-related sequences. In yeast, a *SOK1* protein, sharing sequence homology to a testis-specific mouse gene, suppresses cyclic AMP-dependent protein kinase mutants. Deletions in SOK1 in *Saccharomyces* can lead to an increase in lifespan of 15% or higher [38]. The human homolog to mouse Tcp11 is only expressed in fertile adult testes and is thought to be important in sperm function and fertility [39]. The SOK1 or MST4 family of kinases are known signaling molecules for cell proliferation in multicellular organisms and have been implicated in cancer [40].

Potri.009G066100 (MPK3), a member of a 21-gene family comprised of four groups and is orthologous to the defense-related gene *AtMPK3* [41]. MPKs are generally involved in directing cellular responses to a variety of stimuli, such as osmotic stress and heat shock, and they regulate cell functions, including proliferation, gene expression, differentiation, mitosis, cell survival, and apoptosis [42]. Interestingly, homologs of PtMPK3 in humans have been linked to various forms of cancer [43].

Potri.012G083800, a RPD3 HISTONE DEACETYLASE (RDP3), is present as a single copy gene in *Populus* and is found as co-orthologs in all sequenced plant genomes. Potri.012G083800 shares sequence similarity with two *Arabidopsis RNA-MEDIATED TRANSCRIPTIONAL SILENCING 1* genes (At5g63110 and At5g35600). Histone acetylation/deacetylation, in combination with various MAPKs, has been reported to play a role in plant defense [44]. Histone deacetylases are primarily involved in regulating DNA transcription via modification of histone and chromatin structure and are often implicated in cellular processes such as cell growth, cell cycle and apoptosis. Posttranslational modification of histones has an intriguing but not fully understood role in human cancer [45]. Moreover, histone acetylase PRZ1 in *Arabidopsis* acts as a transcriptional coactivator to modulate auxin effects on gene expression. Whereas auxin promotes formation of lateral roots in wild type, and both auxin and cytokinin are necessary for callus formation, prz1-1 mutants will produce callus in the presence of either auxin or cytokinin [46, 47]. In humans, histone acetylation/deacetylation has been linked to chronic myeloid leukemia. Histone deacetylase has also been reported to impact open chromatin and increase gene expression in pluripotent human cancer cells [48]. We suggest that Potri.012G083800 is a candidate gene for midstream control of signal transduction of cell proliferation in *Populus*.

Potri.018G014800 is a CHLOROPLAST NUCLEIOD DNA-BINDING-RELATED (CNDbr)/Aspartyl protease (Pfam00026) and variants within the GWAS contain a premature stop codon at position Chr18:1196058 bp that is associated with higher callus formation. In tobacco, CNDbr proteins have proteolytic activity and have been shown to bind to DNA [49]. CNDbr proteins have also been linked to leaf senescence[50]. In humans, proteins containing aspartyl protease domains includes the gene encoding Cathepsin D (CTSD), which has been implicated in breast cancer, and the gene encoding Cathepsin E (CTSE), which has been implicated in stomach cancer [51]. Although annotated as a CHLOROPLAST NUCLEIOD DNA-BINDING-RELATED protein, Potri.018G014800 may primarily be related to general cell differentiation.

Several transcription factors, including LEC2, have been implicated in ectopic callus formation in Arabidopsis through transgenic studies. Ikeda and Ohme-Takagi (2014) have implicated WIND1, WUS and TCP as genes that regulate callus formation [52]. LATERAL ORGAN BOUNDARIES DOMAIN (LBD16) transcription factors have also been reported to induce callus formation in *Arabidopsis* [53]. And, ectopic overexpression of OPB4, another transcription factor, resulted in enhanced callus formation in *Arabidopsis* [54]. And, Iwase et al. (2013) successfully overexpressed *AtWND1* to promote callus formation in phytohormone-free medium in tobacco [55]. Surprisingly, none of the orthologs to the transcription factors described above showed significant associations with callus formation in *Populus* in our GWAS analysis. This difference could be related to species-specific differences in regulating and inducing callus, however it is more likely that these differences are due to experimental approach. Our GWAS approach was conducted with no *a priori* assumptions concerning which genes were controlling callus formation, and thus identified only those loci that satisfied the statistical thresholds. The GWAS-identified genes, particularly, SOK1 and MAPK3, may be acting as checkpoints that monitor environmental queues, as discussed above. Such checkpoint genes could be overwhelmed by ectopic regulator expression in *Arabidopsis*. Human cell checkpoint genes are known to sense environmental signals such as ribonucleotide pools or oxygen tension and can lead to tumor formation if mutated [56]. It may also be that the orthologs of those genes tested in *Arabidopsis* did not vary in our population and therefore were not detectable using GWAS approaches. However, there is substantial SNP variation across *Populus* orthologs of these *Arabidopsis* genes (SNP data available at: https://cbi.ornl.gov/data). It is also possible that the *Arabidopsis* orthologs are indeed influencing callus formation in *Populus*, but to a lesser degree than the genes identified in our GWAS test. Ectopic overexpression approaches may overwhelm innate gene and gene network influences on callus formation and impair *de novo* gene discovery. Ectopic overexpression of transcription factors likely leads to perturbations in multiple downstream phenotypes.

In support of *de novo* gene discovery via GWAS approaches, we examined the Affymetrix resource developed for callus induction in *Arabidopsis* and found significant fold change in four orthologs of our eight candidate genes. Interestingly, the two kinases discovered in our study, Potri.003G018500 and Potri.009G066100, display significant negative fold change after 96 hours, while a gene with strong homology to human malignancy, Potri.008G208200, displayed a significant 4-fold change in expression after 96 hours. In further support of *de novo* discovery approaches, the eight genes reported here are significantly co-expressed with genes related to cytokinesis, tubulin, spindle function, and cell differentiation. Our results also show strong connections to genes found in humans related to tumor formation and cancers, suggesting a shared ancestral origin related to the regulation of cell cycle control, cell division and cell differentiation. Birnbaum and Alvarado (2008) have proposed that multi-cellular organisms across the plant and animal kingdoms may be subject to shared mechanisms governing cell regeneration, orchestrated cell differentiation and cell proliferation.

Collectively, our results, and those cited above, support the hypothesis presented by Ikeuchi et al. (2013) on mechanisms of repression and induction during coordinated gene expression required for maintaining normal cell growth and differentiation. The results from our GWAS analysis and the network analysis for our eight candidate genes suggest these genes operate in a tightly regulated manner where some members of the co-expressed gene network have a positive impact on callus formation and others have a negative impact on callus formation. Viewed in this manner, callus formation represents the disruption of the orchestrated regulation of characteristic gene expression that leads to cell differentiation and growth and ultimately tissue and organ formation. Similar promotive and antagonistic networks have been proposed in *Arabidopsis* and in humans.

Finally, while the ratio of exogenous phytohormones plays a role in developing successful protocols for plant regeneration, equally important is the identification of the genetic basis for regeneration capacity of plants. For example, ectopic expression of the transcription factor BABY BOOM in *Arabidopsis* promoted explants regeneration on hormone-free medium and further enhanced regeneration when supplemented with growth regulators [57]. The candidate genes from our GWAS analysis add to the gene inventory for callus formation while our co-expression data, containing known orthologs of callus formation genes, further implicates novel genes that are putatively involved in the pathway. Functional validation will be necessary to investigate the role of these GWAS genes in callus formation and to determine whether or not a callus-formation phenotype will be enhanced with a gene-stacking approach from our correlation networks or in combination with varying phytohormone ratios. These experiments will likely have important implications for adoption of knowledge to develop successful *in vitro* systems for recalcitrant plant species.

## Supporting information

S1 Fig Tissue and organ expression patterns of four callus formation genes and four callus rating score genes. Data was obtained from Phytozome 11.0 (Goodstein et al. 2012) and depicted as log2(FPKM).
(DOCX)

S2 Fig Heat map of differentially expressed *Arabidopsis* orthologs, over 96 hours during callus induction, for *Populus* genes associated with callus formation or callus score in a genome-wide association study. Data taken from: www.ncbi.nlm.nig.gov/geo/ (Fan et al. 2012).
(DOCX)

S3 Fig Co-expression network for orthologs of *Arabidopsis* genes tested in transgenic experiments and their association with *Populus* callus formation and callus rating genes identified via genome-wide association approaches. *Arabidopsis* orthologs are presented in parenthesis and *Populus* candidate genes are underlined. The *Populus* genes were discovered using a GWAS approach; the *Arabidopsis* genes were significantly co-expressed with the candidate genes. Red edges indicate a positive co-expression at r≥0.9 and blue edges indicate negative co-expression at r≤-0.9.
(DOCX)

S1 Table qRT-PCR primers used in the transient protoplast assay. (XLSX)

S2 Table Chromosome location for single nucleotide polymorphisms associations with Populus callus phenotypes that exceeded a Bonferroni-adjusted significance threshold [p = 3.47E−05]. (XLSX)

S3 Table Negatively co-expressed genes between Potri.006G222700, a gene of unknown function, and Potri.008G208200, a RALF-LIKE protein 22. (XLSX)

S4 Table Negatively co-expressed genes between Potri.006G222700, a gene of unknown function, and Potri.012G083800, a RPD3 histone deacetylase protein. (XLSX)

S5 Table Negatively co-expressed genes between Potri.004G118700, a targeting protein for XKLP2, and Potri.015G023600, a gene of unknown function. (XLSX)

S6 Table Co-expressed genes between Potri.004G118700, a targeting protein for XKLP2, Potri.015G023600, a gene of unknown function, and Potri.018G014800, CNDbr, Chloroplast Nucleoid DNA-binding-related gene. (XLSX)

S7 Table Co-expressed genes between Potri.003G018500, a SOK1 kinase, and Potri.012G083800, a RPD3 histone deacetylase protein. (XLSX)

S8 Table Co-expressed genes between Potri.009G066100, a mitogen-activated protein kinase, and Potri.018G014800, CNDbr, a Chloroplast Nucleoid DNA-binding-related gene. (XLSX)
