# Supplementary material for: Defining the genetic components of callus formation: A GWAS approach
Source: PLoS One. 2018 Aug 17;13(8):e0202519. doi: 10.1371/journal.pone.0202519 (PMC6097687; doi:10.1371/journal.pone.0202519)
Supplement: S1 Fig — Data was obtained from Phytozome 11.0 (Goodstein et al. 2012) and depicted as log2(FPKM). (DOCX) [file pone.0202519.s001.docx]

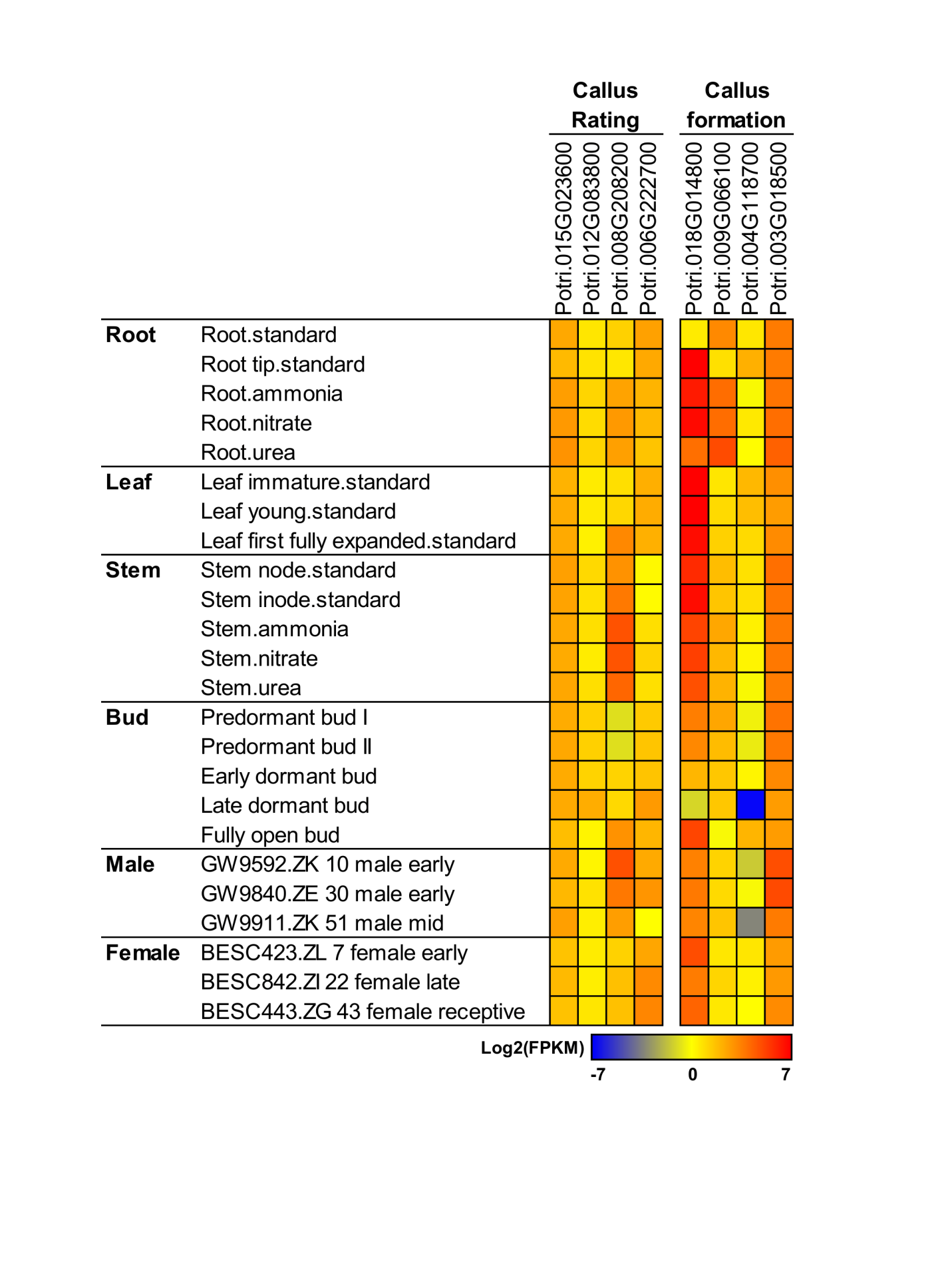


**S1 Fig. Tissue and organ expression patterns of four callus formation genes and four callus rating score genes**. Data was obtained from Phytozome 11.0 (Goodstein et al. 2012) and depicted as log2(FPKM).
