# Supplementary material for: Defining the genetic components of callus formation: A GWAS approach
Source: PLoS One. 2018 Aug 17;13(8):e0202519. doi: 10.1371/journal.pone.0202519 (PMC6097687; doi:10.1371/journal.pone.0202519)
Supplement: S2 Fig — Data taken from: www.ncbi.nlm.nig.gov/geo/ (Fan et al. 2012). (DOCX) [file pone.0202519.s002.docx]

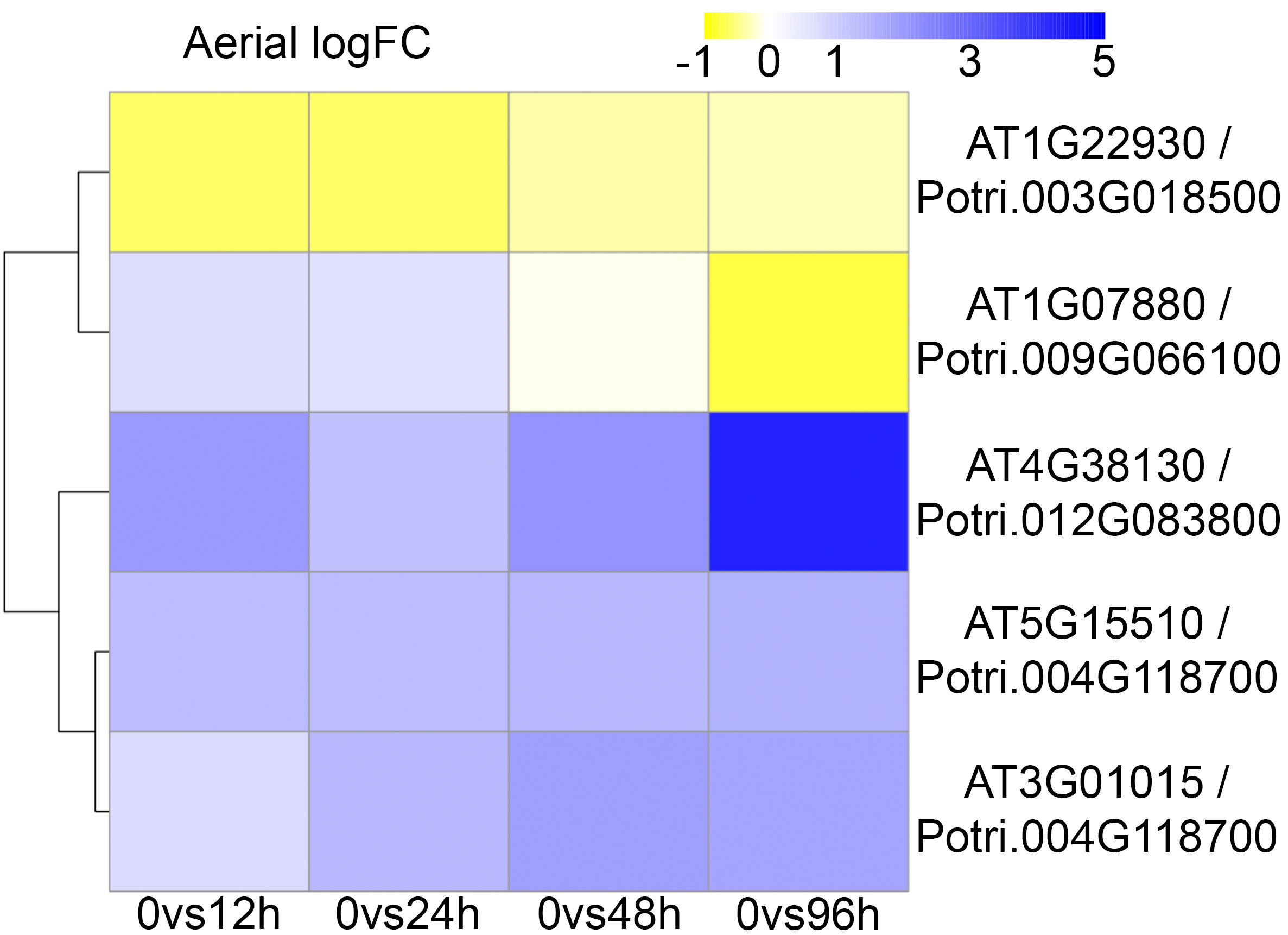


**S2 Fig. Heat map of differentially expressed *Arabidopsis* orthologs, over 96 hours during callus induction, for *Populus* genes associated with callus formation or callus score in a genome-wide association study.** Data taken from: [www.ncbi.nlm.nig.gov/geo/](file:///C:\Users\gtk\AppData\Local\Microsoft\Windows\Temporary%20Internet%20Files\Content.Outlook\RDPWA2DP\www.ncbi.nlm.nig.gov\geo\) (Fan et al. 2012).
