# Supplementary material for: Defining the genetic components of callus formation: A GWAS approach
Source: PLoS One. 2018 Aug 17;13(8):e0202519. doi: 10.1371/journal.pone.0202519 (PMC6097687; doi:10.1371/journal.pone.0202519)
Supplement: S3 Fig — Arabidopsis orthologs are presented in parenthesis and Populus candidate genes are underlined. The Populus genes were discovered using a GWAS approach; the Arabidopsis genes were significantly co-expressed with the candidate genes. Red edges indicate a positive co-expression at r≥0.9 and blue edges indicate negative co-expression at r≤-0.9. (DOCX) [file pone.0202519.s003.docx]

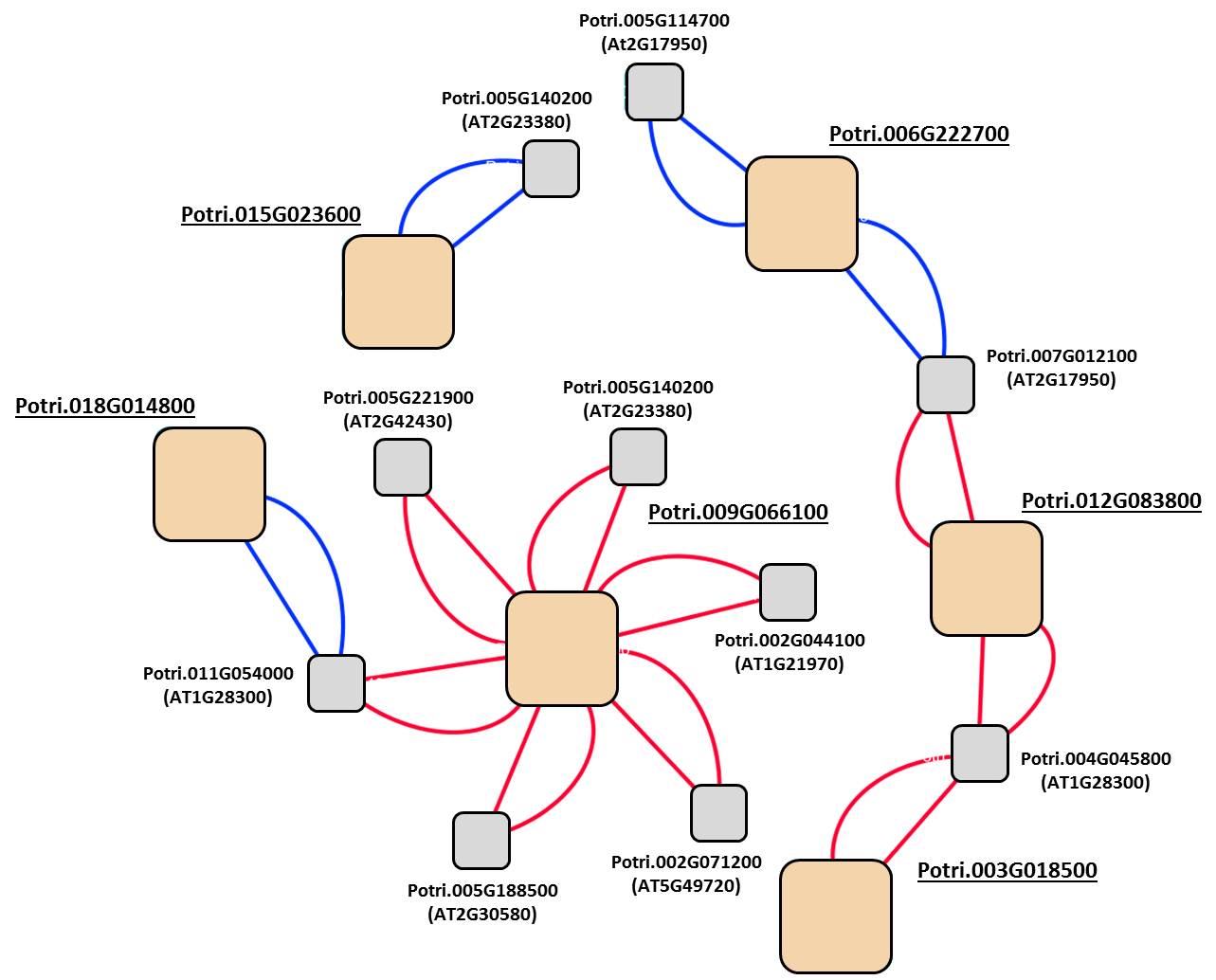


**S3 Fig. Co-expression network for orthologs of *Arabidopsis* genes tested in transgenic experiments and their association with *Populus* callus formation and callus rating genes identified via genome-wide association approaches**. *Arabidopsis* orthologs are presented in parenthesis and *Populus* candidate genes are underlined. The *Populus* genes were discovered using a GWAS approach; the *Arabidopsis* genes were significantly co-expressed with the candidate genes. Red edges indicate a positive co-expression at r>0.9 and blue edges indicate negative co-expression at r≤-0.9.
